# The Role of Predictions, Their Confirmation, and Reward in Maintaining the Self-Concept

**DOI:** 10.3389/fnhum.2022.824085

**Published:** 2022-03-24

**Authors:** Aviv Mokady, Niv Reggev

**Affiliations:** ^1^Department of Psychology, Ben-Gurion University of the Negev, Be’er Sheva, Israel; ^2^Zlotowski Center for Neuroscience, Ben-Gurion University of the Negev, Be’er Sheva, Israel

**Keywords:** predictive processing, belief maintenance, self-concept, motivations, reward, self-verification, self-enhancement

## Abstract

The predictive processing framework posits that people continuously use predictive principles when interacting with, learning from, and interpreting their surroundings. Here, we suggest that the same framework may help explain how people process self-relevant knowledge and maintain a stable and positive self-concept. Specifically, we recast two prominent self-relevant motivations, self-verification and self-enhancement, in predictive processing (PP) terms. We suggest that these self-relevant motivations interact with the self-concept (i.e., priors) to create strong predictions. These predictions, in turn, influence how people interpret information about themselves. In particular, we argue that these strong self-relevant predictions dictate how prediction error, the deviation from the original prediction, is processed. In contrast to many implementations of the PP framework, we suggest that predictions and priors emanating from stable constructs (such as the self-concept) cultivate belief-maintaining, rather than belief-updating, dynamics. Based on recent findings, we also postulate that evidence supporting a predicted model of the self (or interpreted as such) triggers subjective reward responses, potentially reinforcing existing beliefs. Characterizing the role of rewards in self-belief maintenance and reframing self-relevant motivations and rewards in predictive processing terms offers novel insights into how the self is maintained in neurotypical adults, as well as in pathological populations, potentially pointing to therapeutic implications.

## Introduction

Predictive processing is a theoretical framework for understanding the principles guiding human behavior, as illustrated in this special issue (Clark, 2013; Hohwy, 2013; Ueda et al., 2021). The predictive processing (PP) framework posits that people constantly create predictions about the sensory and interoceptive inputs they expect to receive to facilitate their perception of their surroundings. These predictions are then set against the actual input received from the world to create a prediction error (PE), defined as the difference between the predicted and received information. Common PP interpretations assert that perceivers strive to minimize PE to facilitate fluent interaction with their surroundings (Gilead et al., 2020; Hohwy, 2020). To minimize PE, people usually employ one of two methods. The first and more common application of PP principles involves updating the prior beliefs driving the prediction, thus improving the correspondence between future predictions and reality (e.g., Friston et al., 2009; Nassar et al., 2010; Sharot and Garrett, 2016; Vlasceanu et al., 2021; Elder et al., 2021). The second method involves changing the way people perceive reality (“active inference” in PP terms; Friston, 2010; Hohwy, 2020; Yon et al., 2021), for example by reinterpreting incoming inputs to better align with their predictions (e.g., motivated reasoning; Kunda, 1990; Epley and Gilovich, 2016). To date, most PP theories have focused on the belief-updating process, its effects, and PE minimization (e.g., Rao and Ballard, 1999; Friston et al., 2009; Griffiths et al., 2018; Elder et al., 2021; Kube et al., 2021). However, only a handful of contributions have examined how PP can lead to belief-maintaining processes of stable constructs (see, for instance, Gershman and Cikara, 2021). This perspective manuscript suggests that predictive processing principles guide the maintenance of a stable self-concept, whether positive in neurotypical adults or negative in specific pathological populations. To do so, we examine how the PP framework extends prior notions of self-relevant motivations with a novel emphasis on the role of subjective rewards in self-belief maintenance when no PE ensues.

### Predictive Processing and Self-Related Cognition

Principles of PP can explain several stable aspects of personality and social functions that mediate the maintenance of beliefs central to oneself (Yon et al., 2019). For instance, hard-to-falsify religious or supernatural beliefs are often held with very high precision (i.e., individuals attribute substantial weight to these prior beliefs; e.g., Harris and Corriveau, 2021). Such high precision, in turn, leads individuals to interpret inputs in ways that maintain these prior beliefs (i.e., to engage in PE-minimization *via* active inference; van Elk and Aleman, 2017; Gershman, 2019). Similar effects were demonstrated for stereotypical beliefs, whereby seeing an individual who conforms to the stereotype (triggering a small PE) strengthens prior convictions. In contrast, an individual diverging from the stereotype (generating a large PE) can be categorized into a “subtype,” i.e., a member of a subcategory with distinctive features. This subcategory then prevents changing the primary category’s parameters, thus avoiding changing prior beliefs (Kunda and Oleson, 1995; Gershman, 2019; Westra, 2019; Gershman and Cikara, 2021). In the domain of the self, Hohwy and Michael (2017) suggested that people perceive and maintain their self-hood by an internal model of hierarchical endogenous (hidden) causes. The interaction between high-level causes such as desires or long-term goals and low-level causes such as actions generates top-down predictions and minimizes bottom-up PEs. Finally, Moutoussis et al. (2014) go even further to suggest that the predictive brain and Bayesian inference together shape how people understand their own self-concept by taking actions that will most probably fulfill goals of desired self-representations. These studies suggest that, in cases of high-precision beliefs, individuals minimize PE by shaping reality or reinterpreting new inputs to match and maintain these beliefs.

Notably, most implementations of the PP framework to self-relevant judgments have focused either on characterizing the mechanisms supporting belief-updating or on the actions people take to pre-emptively minimize PE (e.g., Moutoussis et al., 2014; Sharot and Garrett, 2016; Kube and Rozenkrantz, 2021). From an evolutionary standpoint, to ensure optimal adaptation to local surroundings, people should indeed be motivated to minimize PE by updating their beliefs in general and their beliefs about themselves in particular (Okasha, 2013). In the current article, we propose that belief-updating is but one of several approaches people employ when encountering self-relevant information. Specifically, we suggest that self-relevant belief maintenance plays an equally important role in shaping one’s cognitions, feelings, and motivations. In the following sections, we briefly describe previous conceptualizations of self-related constructs and explore how a PP framework can apply to processes involving these constructs. We then characterize the role reward plays in maintaining self-relevant beliefs using PP principles. We conclude by discussing the implication of our framework.

### Conceptualization of the Self-Concept and Supporting Motivations

To apply PP principles to the self-concept, we first need to understand its nature and predictive features. Epstein (1973) suggested that individuals continuously gather self-relevant information to gradually construct a “self-theory,” or an inner model from which people make their predictions about themselves. Individuals gather such self-relevant information from various sources, including social (Cooley, 1902; Mead, 1934) and personal (Bem, 1972) cues. Thus, before establishing a stable self-concept, people update their beliefs about themselves according to inputs from their surroundings. Once the theory of the self is consolidated, individuals gradually assign increasing weights to their self-concept to facilitate its maintenance at the expense of continuous updating. Inner models (priors) such as the self-concept and the predictions they make affect, in turn, how people interact with the world and how such interactions affect their previous priors (for a review see Briñol and Petty, 2021). The priors and their precision come into play, for instance, when credible or non-credible sources validate (or invalidate) previous beliefs (Tormala et al., 2006) or when predicting other’s actions (for a review see Bach and Schenke, 2017). Priors also affect the judged fit of a message with one’s goal and situation (Cesario et al., 2004) or the potential fit of a decision with one’s (cued) identity (Oyserman, 2009; Oyserman et al., 2012). In this sense, the nature of self can be seen as a generative model consisting of beliefs (priors) and predictions that, in turn, interact with the world (Hohwy and Michael, 2017; Van de Cruys and Van Dessel, 2021).

Past research has characterized several motivations that govern self-relevant beliefs. According to self-verification, people strive to experience their surroundings and interactions as confirming their self-concepts, thus maintaining their beliefs about themselves (Swann, 2011), in line with a general need for cognitive consistency (Kruglanski et al., 2018). Complementarily, self-enhancement motivates people to pursue positive self-evaluations under the umbrella of people’s general endeavor to feel good about themselves (Taylor and Brown, 1988). Self-enhancement theory suggests that people over estimate their abilities, positive attributes, general self-worth (the above-average effect; Sedikides and Gregg, 2008), as well as the likelihood of positive future events (Sharot, 2011). To satisfy both motivations, people employ various behavioral and interpretive strategies before, during, and after interactions (Swann and Read, 1981). People opt to interact with partners that will provide feedback satisfying their self-motivation (Swann and Read, 1981; Swann et al., 1989, 1994; Burke and Stets, 1999), and, when possible, choose to receive positive information (Sedikides, 1993; Charpentier et al., 2018). Additionally, self-enhancement leads individuals to attribute positive outcomes to the self and negative consequences to external factors, such as other people or situational circumstances (the fundamental attribution error; Ross et al., 1977; Ross, 2018). Accordingly, people allocate more attention to and better recall information according to their motivations (self-verifying; Swann and Read, 1981; Maheshwari et al., 2021; self-enhancing; Sedikides and Green, 2000). In sum, people use coalescing mechanisms to align future inputs (and the interpretations of these inputs) with self-relevant predictions, both for predictions aligned with the self-concept and for more positive predictions.

### The Impact of Self-Relevant Motivations on Predictive Processing

**Figure 1:**
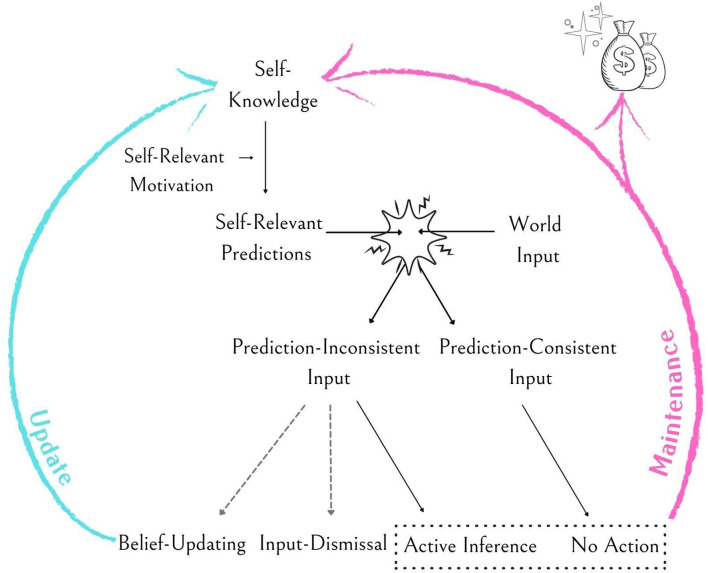
Illustration of the proposed model. Self-Knowledge serves as an inner model of the self (i.e., prior) from which predictions are derived. The (context-dependent) dominant self-relevant motivation, be it self-verification, self-enhancement, or another form of motivation, mediates these predictions. These predictions are compared with inputs from the world, leading to a match or a mismatch. The outcomes of these comparisons lead to one of several possible outcomes. The less likely outcomes (indicated by broken lines in the figure) include model updating (in the unlikely case of belief-updating following prediction-inconsistent information) and input-dismissal (when receiving information that seems extremely irrelevant to the self). More likely outcomes (indicated by solid lines) include self-knowledge maintenance *via* active inference or prediction-consistent input. The more likely outcomes (grouped by the broken lined box) also generate a reward response (money bag), thus further increasing their likelihood to be used in the future.

As highlighted above, individuals constantly rely on their self-concept (i.e., their prior) to predict the inputs and responses of their environment. We suggest that the self-verifying and self-enhancing motivations heavily impact these predictions and the weight given to information pertinent to these predictions (i.e., the input from the world—the evidence; see Figure 1). For example, when the motivation to self-verify dominates, predictions will be consistent with the self-concept; this will be the case even if the relevant self-related features are negative. In contrast, when self-enhancement motivation prevails, predictions will entail evaluations that are more positive and optimistic than the self-concept. When individuals engage in self-relevant interactions, these predictions are compared with incoming inputs. A matching input—self-consistent information for a self-verifying prediction or an input more positive than the self-concept for a self-enhancing prediction—triggers a minimal PE; the information complements prior predictions and thus increases the precision of future predictions. Furthermore, as we elaborate below, we suggest that information congruent with self-relevant predictions triggers a reward response, thus strengthening the strive to minimize PE in the future (see Figure 1).

However, many inputs from one’s environment involve information that mismatches the self-verifying or self-enhancing predictions, thus triggering a PE. Subsequently, perceivers attempt to minimize the PE *via* one of three possible types of reactions. One type of PE minimization prominent in self-verification (Swann, 2011) and self-enhancement theories (Taylor and Brown, 1988) involves maintaining self-beliefs by altering the perception of reality to conform to self-relevant predictions (i.e., active inference). As the self-theory builds on a lifetime of accumulated evidence, the precision of the predictions it creates is typically much higher than that of a new input incongruent with these predictions. Therefore, to explain such incongruent inputs, perceivers employ interpretive strategies to *post hoc* explain how such inputs cohere with the predictions. These strategies include, for instance, developing auxiliary hypotheses (Gershman, 2019) or attributing the input to external situational circumstances that do not call for a model update (e.g., the fundamental attribution error; Campbell and Sedikides, 1999; Ross, 2018). Other PE minimization options involve updating self-beliefs to match the information [in line with Festinger’s cognitive dissonance theory (Festinger, 1957)] or dismissing and ignoring the mismatching event altogether. However, both options are significantly less likely than active inference. To navigate their lives, people need a stable self-concept; frequent updates will result in inefficient use of resources and a high potential for erroneous updates. Similarly, people are unlikely to completely ignore information unless the input is very unlikely (e.g., telling a tall person she is short; Kube et al., 2021). Thus, although all these strategies lead to the end goal of minimizing PE, perceivers are more likely to engage in active inference to solve the PE and maintain prior knowledge in the case of stable constructs such as the self.

### The Reward Value of Confirming Self-Relevant Predictions

Strategies that employ PE minimization can explain many human behaviors, including self-relevant belief maintenance (see, for example, Moutoussis et al., 2014). However, frameworks that emphasize how individuals reckon with PE often overlook what happens when inputs match predictions—when no PE ensues. Typical PP frameworks implicitly assume that when PE equals zero, no action takes place. In contrast, both recent and classic theoretical accounts suggest that individuals strive to maintain a self-consistent worldview (Thorndike, 1911; Festinger, 1957; Theriault et al., 2021)—i.e., to actively keep their PE minimized. For example, people prefer to feel emotions that will maintain a desired (therefore predicted) state, serving a long-term goal (e.g., standing your ground), even if that means feeling an unpleasant emotion (e.g., anger; Millgram et al., 2015; Tamir et al., 2017). Furthermore, people dislike individuals succeeding in stereotype-incongruent roles, both for gender stereotypes (Heilman et al., 2004; Rudman et al., 2012; Moss-Racusin and Johnson, 2016) and ethnic stereotypes (Mendes et al., 2007), leading to negative social interactions and evaluations.

Motivation to maintain a self-consistent perception of the world should go hand-in-hand with the motivation to achieve the goal of a minimal PE. Drawing on classic literature, obtaining a goals hould generate a subjective reward response (Reiss, 2004; Bromberg-Martin and Sharot, 2020). Numerous studies have shown that an event that satisfies an organism’s goal triggers subjective feelings of reward as well as activity in neural structures associated with a reward response such as the ventral striatum and the medial prefrontal cortex (O’Doherty, 2004; Delgado, 2007). Goal achievement triggers a reward response for survival-related goals, such as nutrition and reproduction, and higher-level goals, such as securing resources for future generations and subjective well-being. If maintaining beliefs supporting a predicted world is one’s goal, then the individual holding the beliefs should experience reward whenever they obtain that goal, even when no PE minimization is required. The rewarding experience, in turn, should create a cascade of downstream effects that subsequently reinforces the goal of belief consistency.

Several recent neural and behavioral studies provide initial support for the rewarding aspect of prediction confirmation across domains. Sensory input (in the form of musical sounds) that matches the predicted information triggers an intrinsic reward response (Salimpoor et al., 2015). Similarly, observing targets that match people’s stereotypical predictions is also rewarding (Reggev et al., 2021). From a broader perspective, perceiving information congruent with people’s beliefs, which makes their beliefs more certain, is also rewarding (Bromberg-Martin and Sharot, 2020). These findings suggest that minimal PE (i.e., prediction congruent input) trigger a reward response, highlighting the goal of consistency with predictions. In turn, such reward responses could explain why individuals strive to engage with inputs that have minimal PE, over and above traditional explanations such as fluency (i.e., the ease of processing information; see, for example, Kahl and Kopp, 2017) and free energy (i.e., minimizing “surprise”; Friston, 2010).

If perceiving a minimal-PE input is rewarding in the sensory, social, and cognitive domains, we hypothesize that similar effects should be evident for self-relevant beliefs. Specifically, we suggest that perceiving inputs that produce minimal-PE with self-relevant predictions trigger a subjectively rewarding response. Importantly, we suggest that this reward response can occur regardless of the nature of the specific prediction; a positive input matching a self-enhancing prediction will be as rewarding as a self-consistent input matching a self-verifying prediction. Importantly, we suggest that the reward response for a self-consistent input will occur even if the prediction and its corresponding input are negative. Initial neural findings suggest that self-enhancing processes indeed correlate with reward-related brain regions such as the ventral striatum (Parrish et al., 2022). A similar confirming-related reward can ensue when individuals successfully reinterpret information that initially does not match the prediction, as reinterpreted inputs often conform to the respective predictions. Together, the current framework portrays a vital role for reward and consistency-motivation in people’s tendency to engage with prediction-consistent information and to minimize PE *via* belief-maintaining rather than belief-updating. This role complements the classic PP interpretations that posit that a minimal PE is the end goal of active inference, and thus once it is reached, no more interaction with the stimuli is needed. Additionally, a related reward response may explain why people continue to interact with such information even after the PE is minimized and the prior beliefs and predictions stabilize. Supporting this notion, the value of a reward generated after reaching a desired state has been recently shown to gradually habituate and drive people to actively maintain that desired state to “regenerate” the reward (Dubey et al., 2021). In the context of the current manuscript, after reaching the desired state—a stable self-concept and a predicted world—and experiencing reward caused by prediction confirmation, habituation can kick in and drive people to regenerate reward by reconfirming predictions and thus triggering a reinforcing cycle.

### Implications

Applying reward-oriented processing and PP principles to the motivation to confirm self-relevant predictions offers several exciting implications. First, such an account explains the impact of self-esteem on the balance between self-verification and self-enhancing (Swann and Read, 1981; Taylor and Brown, 1988; Sedikides, 1993; Swann, 2011). People with higher self-esteem tend to self-enhance more frequently and generally expect more positive feedback than people with low self-esteem (Hepper et al., 2011). In contrast, people with low self-esteem experience conflict between wanting to receive positive evaluations (i.e., self-enhancement motivation) and striving for accurate assessments (i.e., self-verification motivation). Typically, such people tend to form accurate-negative predictions and evaluations of essential elements of the self (Ronde and Swann, 1993). Interestingly, as low self-esteem is strongly related to depression (Sowislo and Orth, 2013), people suffering from depression may demonstrate an exacerbated tendency to form negative self-verifying predictions. Recasting previous analyses of depressive automatic thoughts and expectations (Beck, 1963, 1976) in terms of our framework, we go further to suggest that individuals in a state of depression seek inputs consistent with their negative predictions because such inputs trigger rewarding experiences. The reward experience, in turn, may initiate a continuous reinforcement cycle that perpetuates depression and the related phenomenon of depressive realism (Alloy and Abramson, 1979; Moore and Fresco, 2012). If correct, this analysis implies that depression treatment may benefit from examining the reinforcing connection between subjective rewards and evaluations that verify negative predictions or the diminished rewards for positive self-evaluations (see also Van de Cruys and Van Dessel, 2021).

Another field of study that may benefit from these ideas is the study of social anxiety disorder (SAD), a disorder characterized as an ongoing fear of scrutiny by others that will lead to a negative evaluation (Heimberg et al., 2010; Kashdan et al., 2013). At the center of the Cognitive Behavioral Model of Social Anxiety Disorder (Heimberg et al., 2014) lies a “mental representation of the self as seen by others,” i.e., priors based on the self-concept generating predictions of evaluations by others. Indeed, people with SAD see themselves more negatively in social situations and expect interaction partners to see them as such (Kashdan and Savostyanova, 2011). In social interaction, people with SAD allocate attention toward evidence of being evaluated and their flaws (Heimberg et al., 2014) and try to conceal their anxiety by suppressing emotional expression (Butler et al., 2003; Kashdan et al., 2013). For people with SAD, these and additional mechanisms (for a review see: Hofmann, 2007) manifest in most social situations, hindering interactions and contributing to the confirmation of predictions of being perceived negatively. As individuals with SAD keep employing such strategies despite recurrent failures, we propose that the motivation to confirm their predictions (and its associated reward) plays an essential role in maintaining the social anxiety cycle and its underlying negative social self-concept (Van de Cruys and Van Dessel, 2021).

Self-concept maintaining within the PP framework may also explain other social phenomena in which a person has an actively maintained negative or pathological self-concept. For instance, system justification theory (Jost, 2019) posits that socio-economically underprivileged people justify systematic obstacles that maintain the existing social hierarchy and thus their underprivileged position. Our account suggests that the person internalizes her group affiliation as part of her self-concept (similar to Stereotype Threat Theory; Steele, 1992, 1997; Steele and Aronson, 1995). To date, SJT was explained by several joint mechanisms, including the ego, intergroup conflict, and status quo rationalization (Jost et al., 2004). We suggest a more parsimonious explanation building on the predictive self-concept. A reward response to self-concept verification that reifies such underprivileged situations can explain why people act to maintain their current social status, even when it is a disadvantageous one. More broadly, the process of maintaining one’s self-concept *via* the mechanism we suggest here can be applied to many social contexts in which individuals co-opt societal circumstances into their self-concept.

## Conclusion

This perspective exemplifies how the PP framework can be applied to understand the self-concept, emphasizing self-concept maintenance rather than updating. Building on past studies of reward and consistency motives, we suggest that the motivation to maintain rather than update the self-concept manifests as (and is reinforced by) a reward in response to prediction-congruent evidence. Understanding the motives and mechanisms underlying how people perceive themselves may shed new light on behavioral research regarding the self-concept, its development and maintenance, and how it shapes people’s interaction with their surroundings. In addition, it could be key for understanding and changing behaviors characterizing individuals with negative self-views or psychopathological conditions.

## Data Availability Statement

The original contributions presented in the study are included in the article, further inquiries can be directed to the corresponding author/s.

## Author Contributions

AM and NR have conceived the conceptual framework. AM drafted the first version of the manuscript. AM and NR have prepared and edited the manuscript and have approved its final version. All authors contributed to the article and approved the submitted version.

## Conflict of Interest

The authors declare that the research was conducted in the absence of any commercial or financial relationships that could be construed as a potential conflict of interest.

## Publisher’s Note

All claims expressed in this article are solely those of the authors and do not necessarily represent those of their affiliated organizations, or those of the publisher, the editors and the reviewers. Any product that may be evaluated in this article, or claim that may be made by its manufacturer, is not guaranteed or endorsed by the publisher.
